# Pre-operative point-of-care assessment of left ventricular diastolic dysfunction, an observational study

**DOI:** 10.1186/s12871-022-01642-4

**Published:** 2022-04-05

**Authors:** Ylva Stenberg, Ylva Rhodin, Anne Lindberg, Roman Aroch, Magnus Hultin, Jakob Walldén, Tomi Myrberg

**Affiliations:** 1 Department of Surgical and Perioperative Sciences, Anaesthesiology and Intensive Care Medicine, (Sunderbyn), Umeå University, Umeå, Sweden; 2 Department of Public Health and Clinical Medicine, Section of Medicine, Umeå University, Umeå, Sweden; 3 Department of Surgical and Perioperative Sciences, Anaesthesiology and Intensive Care Medicine, (Umeå), Umeå University, Umeå, Sweden; 4 Department of Surgical and Perioperative Sciences, Anaesthesiology and Intensive Care Medicine, (Sundsvall), Umeå University, Umeå, Sweden; 5 Department of Anaesthesiology and Critical Care, Sunderby Hospital, 971 80 Luleå, Sweden

**Keywords:** Diastole, Left ventricular dysfunction, Point-of-care ultrasound, Prospective studies, Risk assessment, Tissue Doppler, Transthoracic echocardiography

## Abstract

**Background:**

Left ventricular (LV) diastolic dysfunction is an acknowledged peri-operative risk factor that should be identified before surgery. This study aimed to evaluate a simplified echocardiographic method using e’ and E/e’ for identification and grading of diastolic dysfunction pre-operatively.

**Methods:**

Ninety six ambulatory surgical patients were consecutively included to this prospective observational study. Pre-operative transthoracic echocardiography was conducted prior to surgery, and diagnosis of LV diastolic dysfunction was established by comprehensive and simplified assessment, and the results were compared. The accuracy of e’-velocities in order to discriminate patients with diastolic dysfunction was established by calculating accuracy, efficiency, positive (PPV) and negative predictive (NPV) values, and area under the receiver operating characteristic curve (AUROC).

**Results:**

Comprehensive assessment established diastolic dysfunction in 77% (74/96) of patients. Of these, 22/74 was categorized as mild dysfunction, 43/74 as moderate dysfunction and 9/74 as severe dysfunction. Using the simplified method with e’ and E/e’, diastolic dysfunction was established in 70.8% (68/96) of patients. Of these, 8/68 was categorized as mild dysfunction, 36/68 as moderate dysfunction and 24/68 as severe dysfunction. To discriminate diastolic dysfunction of any grade, e’-velocities (mean < 9 cm s^− 1^) had an AUROC of 0.901 (95%CI 0.840–0.962), with a PPV of 55.2%, a NPV of 90.9% and a test efficiency of 0.78.

**Conclusions:**

The results of this study indicate that a simplified approach with tissue Doppler e’-velocities may be used to rule out patients with diastolic dysfunction pre-operatively, but together with E/e’ ratio the severity of diastolic dysfunction may be overestimated.

**Trial registration:**

Clinicaltrials.gov, Identifier: NCT 03349593. Date of registration 21/11/2017. https://clinicaltrials.gov.

**Supplementary Information:**

The online version contains supplementary material available at 10.1186/s12871-022-01642-4.

## Introduction

Diastolic dysfunction is a common and often underestimated pathology, referring to abnormalities of left ventricular (LV) relaxation, compliance and filling, regardless of LV systolic function [1–3]. Diastolic dysfunction, particularly if severe, is associated with elevated filling pressures and a reduced preload reserve, thus complicating peri-operative fluid therapy [1]. Higher grade diastolic dysfunction is an acknowledged independent peri-operative risk factor for adverse outcome and should be identified prior to surgery [1, 4–8]. In the pre-operative clinic diastolic function is often unknown, aggravating the risk stratification. Thus, to enable a pro-active and individualized anesthesia management, pre-operative assessment of diastolic function should be conducted in selected cases.

Point-of-care transthoracic echocardiography (TTE) has been shown to have incremental value on clinical management in anesthesiology and intensive care, providing crucial hemodynamic information on cardiac disease and in situ knowledge of volume status [9–11]. However, comprehensive assessment of LV diastolic function is complex as well as time-consuming and may not be realistic to implement in the pre-operative anesthesiologic praxis. In a large systematic review describing the most common pre-operative echocardiographic diagnosis, diastolic dysfunction is not even addressed, highlighting the need of feasible modalities for the purpose [12]. Some recent studies in the field of intensive care and emergency medicine have investigated simplified approaches in order to assess diastolic function rapidly. Tissue Doppler obtained peak velocity of the mitral annulus during early filling (e’) and the ratio of early diastolic velocity of mitral inflow to e’ (E/e’) are two promising measurements [13–16]. However, it has yet to be established if e’-velocities can identify diastolic dysfunction in a pre-operative practice. As follows, the aim of this prospective observational study was to evaluate if a simplified approach with e’-velocities and E/e’-ratio could identify and grade diastolic dysfunction in a pre-operative setting.

## Methods

### Study design and setting

**Fig. 1:**
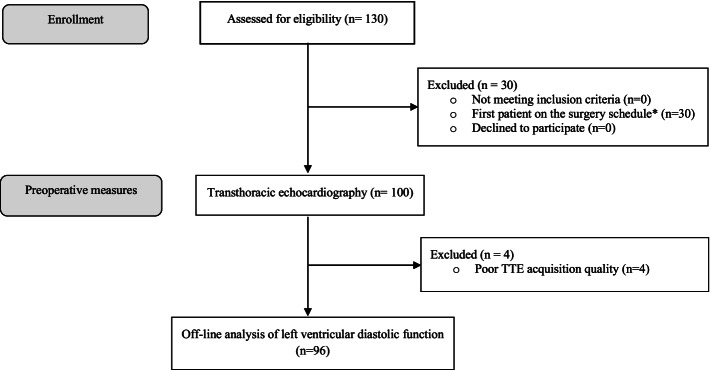
The study flow diagram. *The first patients of each study day were excluded due to the surgery schedule. Abbreviations: TTE = transthoracic echocardiography

This prospective observational study was conducted at a day-surgery unit at a county hospital in Sweden (Sunderby Hospital, Luleå). The collection of data was carried out between 2017 and 12-01 and 2018-11-30. On the study days, when resources were available, 130patients, ≥18 years of age, scheduled for ambulatory surgery were assessed for eligibility for the study. Due to the surgery schedule, the first patients on each study day were excluded (*n* = 30), and 100 patients were included after obtaining written consent. Moreover, patients with severe calcification of the mitral annulus or signs of significant mitral valve regurgitation (i.e., vena contracta ≥4 mm + severe dilatation of the left atrium), arrythmias, i.e., atrial fibrillation/flutter, multiple supraventricular or ventricular extrasystoles in preoperative ECG and/or during TTE scanning and poor acquisition quality in apical four-chamber windows were excluded (Fig. 1).

Ethical approval was provided by the Regional Ethical Board in Umeå, Sweden (Dnr 2016/316–31, December 6, 2016). Informed, signed consent was obtained from all participants. The study was registered prior to patient enrollment at Clinicaltrials.gov (NCT 03349593, principal investigator: Tomi Myrberg, date of registration 21/11/2017). The study adheres to the Strengthening the Reporting of Observational Studies in Epidemiology (STROBE) guidelines.

### Study protocol

**Fig. 2:**
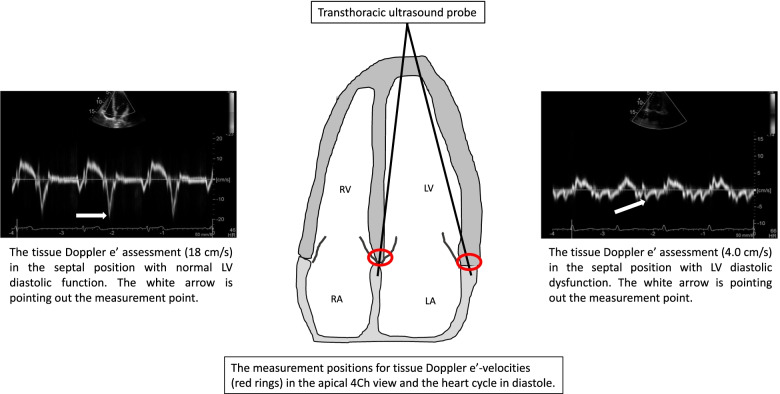
The illustration of the diagnostic test e’. The illustration made by Tomi Myrberg. LA indicates the left atrium; LV, the left ventricle; RA, the right atrium; RV, the right ventricle; TDI e’, tissue Doppler peak velocity of the mitral annulus during early filling of the left ventricle

**Fig. 3:**
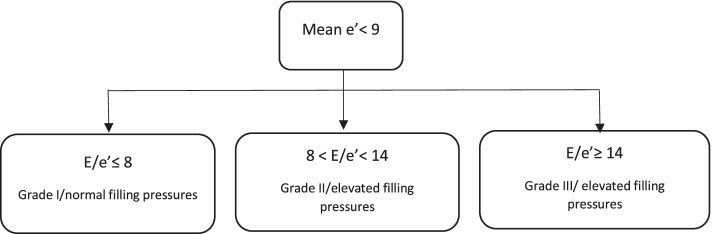
Simplified assessment of diastolic dysfunction by Lanspa et al. Crit Care. 2016;20(1):243. Three categories are established with suspected left ventricular diastolic dysfunction (LVDD) based on mean e’ values and E/e’ ratio: i) e’ < 9 cm s^− 1^ + E/e’ ≤ 8 = LVDD grade I and normal filling pressures, ii) e’ < 9 cm s^− 1^ + E/e’ 8 to 14 = LVDD grade II and elevated filling pressures, and iii) e’ < 9 cm s^− 1^ + E/e’ ≥ 14 = LVDD grade III and elevated filling pressures

The conventional pre-operative fasting period was applied to all patients (i.e., ≥ 6 h nil per os, 200 mL clear liquids allowed 2 h before surgery). All echocardiographic examinations were performed 1–2 h before anesthesia and surgery by a specialist sonographer (LL) or a clinical cardiac physiologist (TM) accordingly to guidelines [17, 18] with GE’s Vivid S70 ultrasound scanner and multifrequency transducer (M5Sc). Data was analyzed off-line using built-in standard software (EchoPac version 113, GE Healthcare, Horten, Norway). Mean values of three consecutive end-expiratory cardiac cycles were recorded for analysis. The automatized GE-software (“auto-EF”) was used to measure biplane left ventricular ejection fraction from the apical 4- and 2-chamber views and categorized into EF ≤40%, 41–49% or ≥ 50%. In the apical 4-chamber view the early (E) and the late (A) transmitral peak velocities and the E/A ratio were measured with pulsed wave Doppler and the Valsalva maneuver was performed. The tissue Doppler mean peak velocity of the mitral annulus (e’) during early filling and peak systolic velocity (Sm) were obtained by tissue Doppler imaging in the septal and lateral walls in the apical 4-chamber view (Fig. 2). E/e’ mean ratio was calculated and a ratio ≥ 14 was classified as increased LV filling pressures. Biplane left atrium volumes were measured and indexed, and tricuspid valve regurgitation was assessed by continuous Doppler. LV diastolic dysfunction was established and graded by the clinical cardiac physiologist (TM) to normal, mild (impaired relaxation = grade I), moderate (pseudonormal = grade II) or severe (restrictive = grade III) [18] using the parameters mean E/e’ ratio, E/A ratio and/or positive Valsalva and/or dilated left atrium in addition to tricuspid regurgitation velocity. No categorization to decreased/normal EF was applied during this diastolic dysfunction grading process. Diastolic dysfunction was also established using a simplified method with e’ and E/e’, similar to the algorithm Lanspa et al. previously presented (Fig. 3). With this method, three categories can be established in suspected left ventricular diastolic dysfunction based on mean e’ values and E/e’ ratio: i) e’ < 9 cm s^− 1^ + E/e’ ≤ 8 = grade I and normal filling pressures, ii) e’ < 9 cm s^− 1^ + E/e’ 8 to 14 = grade II and elevated filling pressures and iii) e’ < 9 cm s^− 1^ + E/e’ ≥ 14 = grade III and elevated filling pressures [13]. Estimation of the right atrium pressure (i.e., level of venous return) was conducted based on the established practice by the maximum size of inferior vena cava (IVCmax) and the inferior cava collapsibility index (IVCCI) [19, 20]. The criterion for signs of hypovolemia was IVCmax < 15 mm + IVCCI > 50% and for hypervolemia IVCmax > 21 mm + IVCCI < 50%. The echocardiographic measurements and reporting adhere to the PRICES checklist (Supplementary file 1) [21].

Patient history was taken, with a focus on dyspnoea and exercise tolerance (metabolic equivalent of task, MET). New York Heart Association (NYHA) Functional Classification was established. Non-invasive blood pressure was measured in a supine position. Blood samples for myocardial damage (high-sensitive troponin I (hs-TnI)) and dysfunction (NT-proBNP) were collected and we classified plasma concentrations for hs-TnI ≥5 ng L^− 1^ [22] respective NT-proBN *P* values > 125 ng L^− 1^ [23] as increased. Point-of-care assessment of LV systolic dysfunction in this cohort was recently published in a separate study [24].

### Statistics

The study data was analyzed with SPSS (IBM Corp. Released 2019. IBM SPSS Statistics for Macintosh, Version 26.0. Armonk, NY: IBM Corp.). The study sample size was calculated to a minimum of 57 individuals for the hypothesized area under the receiver operating characteristic curve at 0.725, null hypothesis at 0.5, a ratio of negative/positive cases at 2, α 0.05, and a power of 0.8 (power = 1-β = 0.8). Moreover, for a comparison of average e’ velocities, the sample size was calculated to overall 56 patients minimum with expected e’ mean velocities for LV diastolic dysfunction at 8.0 cm s^− 1^ and normal LV diastolic function at 11 cm s^− 1^ (SD ± 4, α 0.05 and a power of 0.8). Test efficiency ([Σ true positives + Σ true negatives]/ Σ all cases), sensitivity, specificity, negative predictive value (NPV) and positive predictive value (PPV) were calculated from the cross-tabulations. Levene’s test and Kolmogorov-Smirnov test were applied to analyze the equality of variances respective normality of data. Student’s t test and ANOVA was used to compare mean values, and *p*-values < 0.05 were considered statistically significant. Receiving operating characteristic curves were calculated to determine accuracy and represented as area under the receiving operating characteristic curve (AUROC). The most optimal cut-off of the mean values for e’ to discriminate LV diastolic dysfunction was established by use of Youden’s method (Youden index = sensitivity + specificity − 1). Binominal data was analyzed with Chi-square/Fisher’s exact tests and correlation between the variables reported by the Phi coefficient (φ) when appropriate.

### Intra- and interobservation variability

As a measure of reproducibility, intra- and interobservation variability was controlled based on repeated measurements of 13 randomly selected cases. To calculate intra- and interobservation variability, the equation “variability % = (m_1_–m_2_)/(m_1_+m_2_)/2 x 100%” was used, where m_1_ and m_2_ are mean values of the first and the second measurements of one investigator (TM), or the first measurements between the two investigators (TM and LL).

## Results

After TTE examinations, four patients were excluded due to unusable image quality. Finally, data from 96 patients were used for the analysis (Fig. 1). All patients had a sinus rhythm during TTE scanning. Patient characteristics, symptoms, co-morbidities and medications are summarized in Table 1.

**Table 1:** Patient characteristics, medications and type of surgery in all patients, and comparing those with and without left ventricular diastolic dysfunction established by comprehensive assessment

	***Overall***	***No diastolic dysfunction***	***Diastolic dysfunction***	***p-value***
Number of patients	96	22	74	
Age (years)	63 ± 12	51 ± 13	67 ± 9	< 0.001
Body mass index (kg m^−2^)	27 ± 4	25 ± 3	27 ± 4	0.041
Female sex, n (%)	84 (88)	17 (77)	68 (92)	0.017
Smoker, n (%)	21 (22)	1 (5)	20 (27)	0.025
MET < 4	25 (26)	2 (9)	23 (31)	0.039
ASA-PS III	28 (29)	1 (5)	27 (36)	0.004
NYHA (0)	60 (63)	22 (100)	0 (0)	< 0.001*
NYHA (I)	8 (8.3)	0 (0)	8 (11)	0.192*
NYHA (II)	6 (6)	0 (0)	6 (8)	0.331*
NYHA (III)	22 (23)	0 (0)	22 (30)	0.003*
NT-proBNP (ng L^− 1^)	90 [197]	65 [69]	128 [299]	0.001*
hs-TnI (ng L^− 1^)	6.8 ± 5.2	4.9 ± 2.4	7.4 ± 5.6	0.004
Haemoglobin (g L^L1^)	134 ± 15	139 ± 15	132 ± 14	0.081
Creatinine (μmol L^−1^)	87 ± 93	68 ± 16	92 ± 103	0.316
eGFR (mL/min/L.73m^2^)	70 ± 19	82 ± 10	67 ± 20	0.003
***Co-morbidities***				
Hypertension	67 (70)	11 (50)	57 (77)	0.014
Renal failure	21 (23)	0 (0)	21 (28)	< 0.001
Angina pectoris	14 (15)	0 (0)	14 (19)	0.027
Bronchial asthma	17 (18)	0 (0)	17 (23)	0.013
COPD	12 (13)	1 (5)	11 (15)	0.199
Diabetes mellitus	9 (9)	2 (9)	7 (9)	0.958
***Medications***				
Beta-blockers	33 (34)	2 (9)	31 (42)	0.004
Calcium channel blockers	24 (25)	3 (14)	21 (28)	0.161
ACE- inhibitors	17 (18)	2 (9)	15 (20)	0.228
Angiotensin receptor blockers	20 (21)	2 (9)	18 (24)	0.122
Diuretics	26 (27)	0 (0)	26 (35)	0.001
Nitroglycerine	9 (9)	0 (0)	9 (12)	0.086
Combination therapy*	36 (38)	2 (9)	34 (46)	0.002
***Type of surgery***				
Breast	65 (68)	16 (73)	49 (66)	0.566
Thyroid	16 (17)	3 (14)	13 (18)	0.664
Minor GI	15 (16)	3 (14)	12 (16)	0.770

Values are mean ± SD, number of patients (% of overall) or median and [interquartile range] when appropriate. To evaluate differences between categorical variables Chi2-test or Fischer’s Exact test (*) was used, and for continuous variables student’s T-test or Mann-Whitney U-test (*) was used. Abbreviations: ACE, angiotensin converting enzyme; COPD, chronic obstructive pulmonary disease; GI, gastrointestinal; combination therapy*, beta-blockers, calcium channel blockers, ACE-inhibitors and/or angiotensin receptor blockers, diuretics; ARB, angiotensin receptor blockers; ASA-PS, American Society of Anesthesiologists Physical Status score; NYHA, New York Heart Association functional classification

After comprehensive TTE, diastolic dysfunction of any grade was observed in 77% (74/96) of patients. Of these, 22/74 was categorized as a grade I (mild) dysfunction, 43/74 as a grade II (moderate) dysfunction and 9/74 as a grade III (severe) dysfunction (Tables 2 and 3). Increased filling pressures in LV were found overall in 42/96. Patients with diastolic dysfunction were older compared with those without (mean age 67 ± 9 years versus 51 ± 13 years respectively (*p* < 0.001)) and had to a greater extent ongoing treatment with beta-blockers (*p* = 0.004), diuretics (*p* = 0.001), or combination treatment with beta-blockers, angiotensin-converting enzyme inhibitors and/or angiotensin receptor blockers calcium channel blockers or diuretics (*p* = 0.002). Overall LV ejection fraction and mean arterial blood pressure (MAP) did not differ between the groups (*p* = 0.062 and *p* = 0.229, respectively). NT-proBNP was higher among patients with diastolic dysfunction compared with patients without dysfunction (366 ± 564 ng L^− 1^ vs 82 ± 88 ng L^− 1^, *p* < 0.001). In addition, high-sensitive troponin I values were higher in diastolic dysfunction compared with patients with normal diastolic function (7.4 ± 5.6 ng l^− 1^ vs 4.9 ± 2.4 ng L^− 1^, *p* = 0.004) (Tables 1 and 2).

**Table 2:** Echocardiographic parameters, mean arterial blood pressure, natriuretic peptides, high-sensitive troponin in patients without or with grade 1–3 diastolic dysfunction established by comprehensive and simplified assessment

***Diastolic dysfunction (DD)***	***No DD***	***Grade 1***	***Grade 2***	***Grade 3***	***p-value***
**Comprehensive assessment**					
Number of patients	22 (23)	22 (23)	43 (45)	9 (9)	NA
e’ mean (cm s^−1^)	11.1 ± 3.5	6.0 ± 1.5	6.9 ± 2.0	7.7 ± 2.9	< 0.001
e’ lateral (cm s^− 1^)	12.7 ± 4.5	6.8 ± 2.0	7.7 ± 2.5	9.6 ± 4.6	< 0.001
e’ septal (cm s^− 1^)	9.5 ± 3.2	5.2 ± 1.0	6.2 ± 1.6	5.8 ± 1.6	< 0.001
E/e’ mean	7.3 ± 1.7	11.3 ± 4.6	12.8 ± 4.5	15.4 ± 4.6	< 0.001
E/A ratio	1.2 ± 0.4	0.7 ± 0.1	0.98 ± 0.2	1.5 ± 0.5	< 0.001
IVRT (ms)	71 ± 15	94 ± 15	86 ± 16	79 ± 25	< 0.001
TRV > 2.8 m s^−1^	0 (0)	3 (13.6)	4 (9.3)	4 (44.4)	0.005
LAI (mL m^−2^)	23.9 ± 6.5	30.9 ± 10.6	31.1 ± 9.9	39.8 ± 19.1	0.002
LVEF (%)	58 ± 4	53 ± 10	56 ± 8	51 ± 14	0.081
MAP (mmHg)	104 ± 14	106 ± 12	109 ± 12.4	108 ± 21.7	0.567
Heart frequency/min	68 ± 12	72 ± 14	69 ± 10	72 ± 15	0.539
NT-proBNP (ng L^−1^)	65 [69]	155 [534]	86 [166]	817 [1154]	< 0.001*
hs-TnI (ng L^1^)	4.9 ± 2.4	6.2 ± 3.2	7.8 ± 4.4	12.9 ± 10.9	0.001
**Simplified assessment**					
Number of patients	27 (28.1)	8 (8.4)	36 (38.3)	24 (25)	NA
e’ mean (cm s^−1^)	11.0 ± 3.3	7.6 ± 0.8	6.8 ± 1.3	5.3 ± 1.2	< 0.001
e’ lateral (cm s^−1^)	12.9 ± 4.4	8.8 ± 1.0	7.6 ± 1.8	5.8 ± 1.4	< 0.001
e’ septal (cm s^−1^)	9.2 ± 2.9	6.3 ± 1.2	5.9 ± 1.1	4.9 ± 1.1	< 0.001
E/e’ mean	7.9 ± 2.7	7.4 ± 0.8	10.9 ± 1.5	17.6 ± 4.2	< 0.001
E/A ratio	1.2 ± 4.0	0.9 ± 0.4	0.9 ± 0.2	1.0 ± 0.2	< 0.001
IVRT (ms)	74 ± 15	92 ± 14	86 ± 18	89 ± 19	0.011
TRV > 2.8 m s^−1^	1 (3)	0 (0)	4 (11)	5 (21)	0.163
LAI (ml m^−2^)	27.6 ± 11.9	23.2 ± 8.4	28.9 ± 9.5	36.0 ± 12.3	0.014
LVEF (%)	56 ± 8	56 ± 8	55 ± 8	55 ± 10	0.986
MAP (mmHg)	99 ± 15	110 ± 5	109 ± 14	111 ± 10	0.006
Heart frequency (min^−1^)	68 ± 11	69 ± 11	71 ± 12	68 ± 8	0.512
NT-proBNP (ng L^1^)	65 [71]	75 [200]	98 [198]	198 [560]	0.007*
hs-TnI (ng L^−1^)	5.5 ± 4.5	4.9 ± 3.4	7.2 ± 5.4	8.3 ± 5.6	0.151

Values are mean ± SD, number of patients (% of overall) or median and [interquartile range] when appropriate

*Abbreviations*: *IVRT* left ventricular isovolumic relaxation time, *LVEF* left ventricular ejection fraction, *hs-TnI* high-sensitive troponin I, *LAI* left atrial volume index, *MAP* mean arterial blood pressure, *NA* not applicable, *NT-proBNP* N-terminal prohormone of brain natriuretic peptide, *TRV* tricuspid regurgitation velocity

*P* values are ANOVA, Kruskal-Wallis test (*) or Chi-Square test

**Table 3:** Comprehensive vs. simplified assessment for the classification of diastolic dysfunction, *n* = 96

Diastolic function	Comprehensive assessment	Simplified assessment (e’ + E/e’)
No DD	22 (22.9)	27 (28.1)
Grade I DD	21 (21.9)	8 (8.4)
Grade II DD	44 (45.8)	36 (38.3)
Grade III DD	9 (9.4)	24 (25)
Uncategorized	0 (0)	1 (1)*

Values are number of patients (% of overall). Abbreviations: DD, diastolic dysfunction. *e’-velocities missing for one patient

**Fig. 4:**
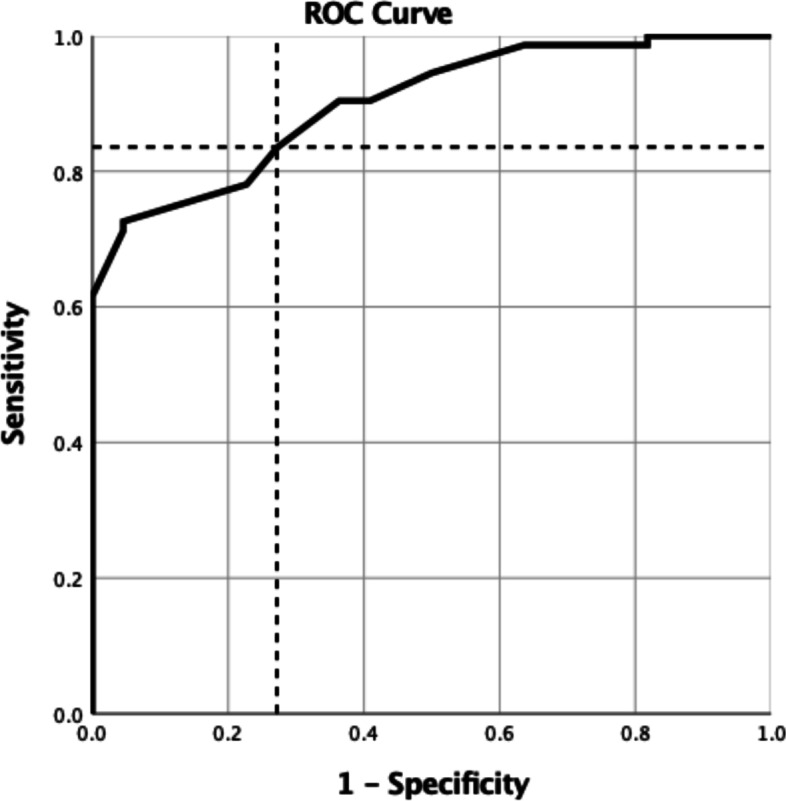
The ROC curve for e’ mean velocities to discriminate diastolic dysfunction. AUROC (95%CI): 0.901 (0.840–0.962), *p* < 0.001. The dashed lines are pointing out the cut-off value 8.75 cm s^− 1^

Using the simplified method (Fig. 3), diastolic dysfunction was identified in 70.8% (68/96) of patients. Of these, 8/68 was categorized as a mild dysfunction, 36/68 as a moderate dysfunction and 24/68 as a severe dysfunction (Table 3). Patient characteristics and echocardiographic data are summarized and compared to the comprehensive assessment in Table 2. The AUROC for mean e’-velocities to identify any grade of diastolic dysfunction was 0.901 (95%CI 0.840–0.962) (Fig. 4). The most accurate cut-off for mean e’ -velocities was 8.75 cm s^− 1^, defined by combination of sensitivity and specificity (i.e., the highest Youden index). At the cut-off value < 9 cm s^− 1^, mean e’-velocities had a sensitivity of 82.2%, a specificity of 72.7%, a PPV of 55.2% and NPV of 90.9% to recognize patients with diastolic dysfunction (Table 4). A moderate correlation was found between e’ < 9 cm s^− 1^ and with overall diastolic dysfunction (φ = 0.530, *p* < 0.001), and mild correlation with symptomatic diastolic dysfunction (φ = 0.266, *p* = 0.010). No correlation could be found between e’ < 9 cm s^− 1^ and asymptomatic diastolic dysfunction (φ = 0.185, *p* = 0.072) or with low exercise capacity (MET < 4), φ = 0.175, *p* = 0.088). e’-velocities alone could discriminate patients with diastolic dysfunction (*p* < 0.001), but not between different grades of diastolic dysfunction (*p* = 0.198).

**Table 4:** Efficiency and accuracy for tissue Doppler e’-velocities to discriminate patients with any grade of LV diastolic dysfunction

***Method***	***Efficiency***	***PPV (%)***	***NPV (%)***	***Sensitivity (%)***	***Specificity (%)***	***AUROC (95%CI)***
e’ (cutoff < 9 cm s^−1^)	0.79	55.2	90.9	82.2	72.7	0.901 (0.840–0.962)

Efficiency, PPV, NPV, sensitivity and specificity were calculated from cross-tabulations

*Abbreviations*: *AUROC* area under the ROC curve, *CI* confidence interval, *NPV* negative predictive value, *PPV* positive predictive value

In addition, mean e’ velocities and mean E/e’ ratio ability to discriminate lower grade of diastolic dysfunction (normal and mild) from higher grade (moderate and severe) were AUROC 0.615 (95%CI 0.500–0.731, *p* = 0.053) and AUROC 0.781 (95%CI 0.688–0.875, *p* < 0.001), respectively.

### Volume dependence

Overall, 43.7% (42/96) of patients had signs of hypovolemia based on the inferior vena cava assessment (i.e., IVCmax < 15 mm + IVCCI > 50%). In this study cohort, there was no difference in prevalence of hypovolemic state between patients with or without diastolic dysfunction, *p* = 0.854. Of those with higher grade of diastolic dysfunction (i.e., moderate and/or severe, *n* = 52), 21/52 (40%) were hypovolemic. There was no difference in tissue Doppler e’-velocities between patients without and with hypovolemia (euvolemia (*n* = 45); hypervolemia (*n* = 7)) and hypovolemia (*n* = 42), (8.1 ± 3.4 cm s^− 1^ vs 7.3 ± 2.5 cm s^− 1^, *p* = 0.194). In addition, no difference in e’-velocities were observed between euvolemia and hypovolemia, or euvolemia and hypervolemia (8.0 ± 3.5 cm s^− 1^ vs 7.3 ± 2.5 cm s^− 1^, 8.0 ± 3.5 cm s^− 1^ vs 8.8 ± 3.4 cm s^− 1^, *p* = 0.371 and *p* = 0.223, respectively).

### Intra- and interobservation variability

The intra- and interobservation variability for this cohort has been published in a previous study [24]. The inter-observation and intra-observation variability (%) for e’ were 4.2 ± 5.9 (95%CI 0.6–7.8) and 0.9 ± 1.8 (95%CI 0.2–2.0), respectively.

## Discussion

This prospective observational study investigated a simplified method with e’ and E/e’ for assessing diastolic function, and the results showed that regardless of which method used (comprehensive or simplified assessment), a high prevalence of diastolic dysfunction was found. Patients with comorbidities including hypertension, ischemic heart disease, chronic kidney disease, bronchial asthma, as well as patients with increased age, low exercise capacity, and smoking were more likely to have diastolic dysfunction. This is in line with previous reports exploring risk factors for diastolic concerns [3]. The proportion of patients with diastolic dysfunction was higher than expected. In a general population, the prevalence of diastolic dysfunction has been reported to approximately 25–27% [2, 3], in contrast to in this unselected ambulatory surgical cohort in which majority of patients presented with diastolic dysfunction. However, this surgical population is not fully representative of the general population in terms of age and comorbidities, and we believe this somewhat unexpected finding may reflect on the high number of female patients with cardiovascular comorbidity such as hypertension and IHD. Nonetheless, the results demonstrate that the simplified method using e’-velocities had a high NPV, sensitivity, and AUROC, indicating that this approach may be used to rule out patients with diastolic dysfunction with an appropriate accuracy.

Furthermore, the results are in line with previous studies and indicate that e’-velocities with a cut-off at 9 cm s^− 1^ may be used to discriminate both symptomatic and asymptomatic patients with diastolic dysfunction from patients without these concerns [13, 14]. However, the poor PPV and rather low specificity may allow for misdiagnosis in terms of confirming diastolic dysfunction and a test result indicating diastolic dysfunction should be interpreted with caution, and always in a clinical context. Additionally, in this cohort e’-velocities alone could not differentiate between different grades of diastolic dysfunction, which from an anesthesiologist point of view is a clear shortage since different patterns of diastolic filling may require different vigilance and strategies. Hence, in order to with certitude diagnose diastolic dysfunction and to discriminate between different grades of diastolic dysfunction a more comprehensive, but indeed more time consuming, assessment may be required.

Regarding the use of E/e’ for assessing filling pressures, previous evidence is somewhat conflicting [15, 25]. In this study, E/e’ performed acceptable in order to discriminate between patients with a normal or mildly reduced diastolic function versus patients with a moderate-severe diastolic dysfunction. However, the simplified method seems to, compared with comprehensive echocardiography, exaggerate the severity of diastolic dysfunction. Nonetheless, point-of-care assessment is a real-time and goal-oriented modality not intended to replace a comprehensive assessment [26], but to present a rapid tool for assessing cardiac function for non-cardiologists when hemodynamic instability and need of volume replacement can be expected, and cardiologist referral may not be suitable. Furthermore, tissue Doppler measurements are acknowledged to be relatively non-volume-dependent [27], supported by the results also in this study where no differences in e’-velocities were found between patients in different volume states. Additionally, e’-velocities per se are reliable in atrial fibrillation [27] and fast to measure in a 4-chamber projection. The time needed for tissue Doppler measurements in the point-of-care context is within 1 min, as shown in our previous study [24].

Diastolic dysfunction is initially often asymptomatic, but is still associated with an increased morbidity and all-cause mortality [2], adverse surgical outcome [4–6, 28], mortality in septic patients [29–31] and weaning failure [32]. On the other side, in a recently published retrospective study on the prevalence of diastolic dysfunction and postoperative outcomes, no significant association was found between diastolic dysfunction and in-hospital mortality or acute kidney injury [33]. In another recent study no association between diastolic dysfunction and need of intra-operative norepinephrine was observed [34]. Notwithstanding, patients with higher grade diastolic dysfunction have an impaired capacity to manage haemodynamic alterations and are at an increased peri-operative risk [1]. Notably, in this cohort almost a half of the patients with higher grades of diastolic dysfunction had signs of low venous return, something that may challenge the perioperative fluid therapy. Thus, for elderly and/or patients with risk factors or symptoms, especially in intermediate or high-risk surgery, pre-operative echocardiographic screening conducted by anesthesiologists would allow for a proactive anesthesia plan to minimize intra-operative hemodynamic instability and perioperative complications. Implementation of a revised pre-operative risk assessment including anesthesiologist-performed TTE to screen for the most common and/or serious cardiac pathology (e.g. LV diastolic and systolic dysfunction, major valvular disease, hypertrophic obstructive cardiomyopathy and severe hypovolemia) may face several challenges [12, 35, 36], but might be included in the concept of peri-operative surgical home introduced recently [35, 37]. Availability of equipment, theoretical knowledge, expert supervision, and training in both simulator and practice are all essential factors for a successful implementation [38]. However, it ought to be possible with a robust educational plan [35, 39–41].

### Limitations

This single-centered observational study had a rather small sample size and despite a consecutive unselected enrollment, a vast majority of the included patients were women (partially explained by a high amount of breast cancer surgery in the hospital). This may have affected the prevalence of diastolic concerns but should not have a negative effect on the assessment of the evaluated methods per se. Larger, multi-centered studies evaluating e’ and E/e’ as a point-of-care assessment of diastolic dysfunction and the association with post-operative outcome, are warranted. All examinations were conducted by expert sonographers to minimize diagnostic bias and a high reproducibility was obtained. In addition, only four patients were excluded due to unusable image quality. The accuracy of evaluated tests may decrease with a less experienced observer, and this may decrease the applicability of the study results. In this study, the diagnosis and grading of diastolic dysfunction were conducted with the recommended parameters from 2009 American Society of Echocardiography (ASE) guidelines [18], with the exception of deceleration time, and the results are based on this data. The 2016 ASE guidelines [27] brings a substantial number of patients with indeterminate diastolic function (Supplementary file 2) and were not used in this study. High-sensitive troponin I may be used for risk stratification at a cut of level of < 5 ng L^− 1^, i.e., well below the concentration used to diagnose myocardial infarction [22, 42]. Most of the patients in the current study had a level ≥ 5 ng l^− 1^, but the study sample size was too small to evaluate significance of hs-TnI for cardiac risk assessment. In addition, the aim of this pre-operative study was to evaluate the accuracy of e’ and E/e’ in order to identify and grade diastolic dysfunction, and no intra- and postoperative data were analyzed. Hence, association between e’-velocities and/or E/e’ and intra-operative hemodynamic stability and postoperative outcome was not assessed in this study.

## Conclusion

The results of this study indicate that a simplified approach with tissue Doppler e’-velocities may be used to rule out patients with diastolic dysfunction pre-operatively, but together with E/e’ ratio the severity of diastolic dysfunction may be overestimated.

## Supplementary Information

**Additional file 1: Supplementary file 1.** PRICES checklist.

**Additional file 2: Supplementary file 2.** Classification of diastolic dysfunction with two guidelines and simplified method.

## Data Availability

The datasets used and/or analysed during the current study are available from the corresponding author on reasonable request.

## Abbreviations

AUROC: Area under the receiver operating characteristic curve
CI: Confidence interval
COPD: Chronic obstructive pulmonary disease
DD: Diastolic dysfunction
EF: Ejection fraction
eGFR: Estimated glomerular filtration rate
ESC: European Society of Cardiologists
e’: tissue Doppler peak velocity of the mitral annulus during early filling
GI: Gastrointestinal
hs-TnI: high-sensitive troponin I
LV: Left ventricular
LVEF: Left ventricular ejection fraction
IVRT: Left ventricular isovolumic relaxation time
MAP: Mean arterial blood pressure
MET: Metabolic equivalent of task
NPV: Negative predictive value
NT-proBNP: N-terminal prohormone of brain natriuretic peptide
NYHA: New York Heart Association
PPV: Positive predictive value
PW: Pulsed wave Doppler
SD: Standard deviation
STROBE: Strengthening the Reporting of Observational Studies in Epidemiology
TDI: Tissue Doppler Imaging
TTE: Transthoracic echocardiography
