# Supplementary material for: Pre-operative point-of-care assessment of left ventricular diastolic dysfunction, an observational study
Source: BMC Anesthesiol. 2022 Apr 5;22:96. doi: 10.1186/s12871-022-01642-4 (PMC8981659; doi:10.1186/s12871-022-01642-4)
Supplement: Supplementary file 1 — Additional file 1: Supplementary file 1. PRICES checklist. [file 12871_2022_1642_MOESM1_ESM.pdf]

	Checklist items	LV systolic function	RV function	LV diastolic function	Fluid management
A1	**Research vs clinical study**				
	• Research study			X	
	• Clinical study			X	
A2	**Study information**				
	• Specific study type			X	
	• State study design			X	
	• Report sample size			X	
A3	**Patient information**				
	• Age			X	
	• Gender			X	
	• Height & weight (or BMI)			X	
	**Comorbidities**				
	• Ischaemic heart disease			X	
	• Atrial fibrillation			X	
	• Hypertension			X	
	• HFpEF			X	
	• HFrEF			X	
	• Pacemaker implant present			X	
	• COPD or pulmonary hypertension			X	
	• CKD or hemodialysis			X	
A4	**Echocardiography information**				
	• Type of echo (TTE or TEE)			X	
	• Indicate if data collected at end-expiration			X	
	• No. of beats used for averaging			X	
	• Report vendor of ultrasound machine			X	
	• Indicate if airway pressure trace displayed on screen				
A5	**Clinical information at the time of echo**				
A5.1	***Ventilation***				
	• Mode of ventilation				
	• Tidal volume				
	• Plateau pressure				
	• PEEP				
A5.2	***Hemodynamics***				
	• Cardiac rhythm & heart rate			X	
	• BP			X	
	• Inotropes, vasopressors and doses				
A6	**Reliability** (for research study)				
	• Feasibility of echo stated			X	
	• Intra-observer variability			X	
	• Inter-observer variability			X	
	• Indicate if observer blinded to treatment, if applicable				
A7	**Statistics** (for research study only)				
	• Sample size calculation			X	
	• Indicate if statistician blinded to treatment / group				
	• Address confounders, if applicable				
	• Internal validation provided, if applicable			X	

Fig. 2 PRICES utility checklist

LV systolic function	B1	**LV systolic function indices**	
		• LV ejection fraction	X
		• Tissue Doppler S' velocity	
		• Mitral annular systolic plane excursion (MAPSE)	
		• LV strain or strain rate	
	B2	**LV size**	
		• LV end-diastolic diameter or volume	
	B3	**Other functional indices to aid interpretation**	
		• Cardiac output	
		• Stroke volume	
		• Any heart valve dysfunction	X
RV systolic function	C1	**RV systolic function indices**	
		• Tricuspid annular systolic plane excursion (TAPSE)	
		• RV fractional area change	
		• Tissue Doppler S' velocity	
		• LV strain or strain rate	
	C2	**RV size and wall thickness**	
		• RV end-diastolic diameter or area	
		• RV:LV end-diastolic area ratio	
		• RV wall thickness	
	C3	**Other functional indices to aid interpretation**	
		• PFO or other shunt(s)	
		• Pericardial effusion	
		• Paradoxical septal motion	
		• Inter-atrial septal bowing	
		• IVC diameter	
LV diastolic function	D1	**Indices for evaluation of LV diastolic function**	
		• E/A ratio	X
		• Tissue Doppler E' velocity	X
		• E/E' ratio	X
		• PAP or TR peak velocity	X
		• LA size	X
		• Mitral E deceleration time	
		• Pulmonary venous flow	
	D2	**Other functional indices to aid interpretation**	
		• BP: systolic, diastolic and mean	X
		• Related chronic medications	X
	D3	**Criteria used for grading diastolic function**	
		• State or quote criteria	X
		• Cite reference	X
		• Technical details of measurements	X
Fluid management	E1	**Evaluation of fluid management**	
		• Define the meaning of FR clearly	
		• State parameter(s) used for predicting fluid responsiveness (FR)	
		• Describe parameters used to assess FR (e.g. cut-offs)	
	E2	**Other information to aid interpretation** (research study only)	
		• State reference standard used in diagnostic or validation study	
		• State if echo is used to measure the reference value (e.g. CO)	
		• State technical information on echo measurements	
		• Describe any procedures used for FR assessment	

Fig. 2 continued
