# Supplementary material for: Pre-operative point-of-care assessment of left ventricular diastolic dysfunction, an observational study
Source: BMC Anesthesiol. 2022 Apr 5;22:96. doi: 10.1186/s12871-022-01642-4 (PMC8981659; doi:10.1186/s12871-022-01642-4)
Supplement: Supplementary file 2 — Additional file 2: Supplementary file 2. Classification of diastolic dysfunction with two guidelines and simplified method. [file 12871_2022_1642_MOESM2_ESM.pdf]

Supplementary file 2. Classification of diastolic dysfunction with two guidelines and simplified method.

Diastolic function	ASE 2016	ASE 2009	e’ + E/e’
No DD	48 (50)	22 (22.9)	28 (29.5)
Grade I	13 (13.5)	21 (21.9)	8 (8.4)
Grade II	15 (15.6)	44 (45.8)	37 (36.5)
Grade III	1 (1)	9 (9.4)	24 (25)
Indeterminate	18 (18.8)	0 (0)	1 (1)
Total	96	96	96

ASE = American Society of Echocardiography. DD = diastolic dysfunction.
